# Self-supervised learning enhances accuracy and data efficiency in lower-limb joint moment estimation from gait kinematics

**DOI:** 10.3389/fbioe.2025.1633513

**Published:** 2025-09-24

**Authors:** Yifan Li, Jiayu He, Bernard Liew, David S. Hollinger, Qichang Mei, Behnam Gholami, Maria Fasli, Klaus McDonald-Maier, Xiaojun Zhai

**Affiliations:** ^1^ Department of Engineering, King’s College London, London, United Kingdom; ^2^ School of Sport, Rehabilitation, and Exercise Sciences, University of Essex, Colchester, United Kingdom; ^3^ School of Computer Science and Electronic Engineering, University of Essex, Colchester, United Kingdom; ^4^ Faculty of Sports Science, Ningbo University, Ningbo, China

**Keywords:** joint moment estimation, self-supervised learning (SSL), data scarcity, data efficiency, lower limb dynamics

## Abstract

**Objective:**

Deep learning (DL) has introduced new possibilities for estimating human joint moments - a surrogate measure of joint loads. However, traditional methods typically require extensive synchronised joint angle and moment data for model training, which is challenging to collect in real-world applications. This study aims to improve the accuracy and data efficiency of knee joint moment estimation via leveraging self-supervised learning techniques to automatically extract human motion representations from large-scale unlabeled joint angle datasets.

**Method:**

We proposed a joint moment estimation method based on self-supervised learning (SSL), using a Transformer auto-encoder architecture. The model was pre-trained on large-scale unlabeled joint angle data with masked reconstruction to effectively capture spatiotemporal features of human motion. Subsequently, we fine-tuned the model using a small amount of labeled joint moment data, enabling accurate mapping from joint angles to joint moments. We evaluated this method on a dataset of 55 normally developing children and compared the performance of the pre-trained SSL model fine-tuned with different amounts of labeled data to a baseline model.

**Results:**

The Fine-tuned model significantly outperformed the baseline model, especially in scenarios with scarce labeled data. MSEs were reduced from 24.00% to 45.16% (with an average reduction of 36.29%), and MAE from 18.18% to 37.80% (with an average reduction of 26.48%). The proposed SSL model exceeded the performance of the baseline model trained with 100% data, using only 20% of the data in the labeled dataset during fine-tuning. When both models were fine-tuned using only 5% of the labeled data, the proposed SSL achieved four-fold better performance than the baseline model.

**Conclusion:**

This study demonstrates that self-supervised learning significantly improves the accuracy and data efficiency of joint moment estimation, providing a more efficient solution for biomechanical evaluation. The proposed model can reduce the burden of collecting data and expand clinical applications.

## 1 Introduction

Joint moments estimation plays a central role in biomechanics and rehabilitation engineering. It is widely applied in the evaluation of musculoskeletal dynamics, with applications ranging from injury prevention to rehabilitation monitoring. Analysis of lower limb joint moments is particularly critical, as it provides insight into load distribution and joint stability, which are essential for diagnosing gait abnormalities and optimizing therapeutic interventions. For example, knee moment is a critical metric for assessing knee joint health (Wang et al., 2020a; Ulrich et al., 2023). Reducing knee adduction moment (KAM) without increasing knee flexion moment (KFM) may alleviate knee symptoms in individuals with knee osteoarthritis (Edd et al., 2020).

Traditionally, the calculation of joint moments requires data from two input sources - marker trajectories of the modeled body segments, and ground reaction forces (Alkjaer et al., 2001). Limitations associated with traditional motion capture include: 1) the need for expensive equipment, 2) the time-consuming nature of data collection and processing, and 3) restricting data collection to within laboratory conditions. Recently, a greater number of studies have shown that joint moments can be predicted based solely on kinematic inputs, such as those measured using an inertial measurement unit (IMU) (Altai et al., 2023; Hossain et al., 2023; Sharifi Renani et al., 2021) by leveraging machine (ML) and deep learning (DL) algorithms. Coupling ML and DL with biomechanical inputs has the strong potential to bring biomechanical assessments from the laboratory to free-living clinical and community environments.

DL algorithms are particularly attractive as a method for predicting joint moments as they do not require predefined assumptions about the system, and can handle complex non-linear relationships and high-dimensional data (Paaß and Hecker, 2023). Among DL models, Deep Neural Networks (DNNs), Convolutional Neural Networks (CNNs), and Long Short-Term Memory (LSTM) networks have demonstrated superior performance in joint moment estimation (Mansour et al., 2023), compared to traditional regression-based models and physics-based biomechanical models, which often rely on hand-crafted features and predefined assumptions. Gait kinematics such as angles (Zhang et al., 2022b), velocities, and accelerations (Xiang et al., 2024), have been widely recognized as effective input features for DL models in predicting joint moments. Kinematic data can be collected using wearable devices, such as an IMU (Uhlrich et al., 2022) and markerless motion capture, enabling real-world, out-of-lab assessment of joint mechanics (Palucci Vieira et al., 2024). However, a critical limitation of DL, and indeed ML, algorithms is the requirement for large labeled datasets. Such labeled datasets are very rarely available because of the time-consuming nature of the collection and processing of motion capture data. For example, it is much easier to collect larger quantities of “unlabeled” kinematic data, such as from IMUs or markerless motion capture, from a greater number of participants in free-living environments, compared to traditional motion capture data.

Training DL models using large-scale datasets has emerged as the mainstream approach (Nguyen et al., 2019; Wang et al., 2020b; Shen et al., 2023). Self-supervised learning (SSL) has emerged as a groundbreaking paradigm to address the challenge of labeled data scarcity in machine learning (Gündüz et al., 2023; Khowaja et al., 2023). By pre-training models on large volumes of unlabeled data, which can later be fine-tuned on a smaller set of labeled data for solving specific tasks, SSL enables models to uncover intricate feature representations and patterns within the data, significantly enhancing their predictive accuracy and generalization capabilities (Hestness et al., 2017; Giakoumoglou and Stathaki, 2024). The pre-training step involves masking certain portions of the input data and then training a model to reconstruct the masked parts based on the remaining visible information (Kenton and Toutanova, 2019). This approach has proven highly effective in various domains, including image processing (Scanvic et al., 2023), natural language processing (NLP) (Kenton and Toutanova, 2019), as well as time-series analysis (Zhang et al., 2024). Recent studies have begun to explore SSL’s potential in biomechanics, from various biomedical signals (Del Pup and Atzori, 2023) to the geometric representations of limb shapes (Gu et al., 2021; Tan et al., 2024). Despite the remarkable success SSL has demonstrated, biomechanical datasets, especially those involving gait kinematics, typically encompass multiple walking speeds and subjects from various age groups, posing challenges for SSL methods based on masking and reconstruction. It remains unclear whether SSL with masking and reconstruction can effectively learn joint angle representations with these complex combined features and enhance performance on downstream tasks of estimating joint moments. Furthermore, predicting joint moments from joint angles remains a challenging and uncertain task.

This paper aims to apply SSL to improve the accuracy and data efficiency of joint moment estimation by utilizing unlabeled joint angle data with a two-stage approach (Table 1). The gait analysis dataset includes joint angle data for 18 lower limb kinematic features. We first pre-trained a transformer-based model on unlabeled joint angle data using masking and reconstruction tasks to extract spatiotemporal features. The pre-trained model was then fine-tuned for a supervised task mapping joint angles to the corresponding joint moments using labeled data. We assessed the SSL model’s performance and data efficiency by comparing it with baseline Transformer models trained from scratch under identical hyperparameter and training configurations as the pre-trained SSL model. Evaluation metrics, including mean squared error (MSE) and mean absolute error (MAE), were used to quantify the differences between predicted and observed joint moments. We selected the hip, knee, and ankle joints as the primary lower-limb joints for evaluating model performance. Furthermore, we compared model accuracy under varying amounts of labeled data. To explore prediction improvements under different input-output scenarios, we utilized each of the three joint angles as input to estimate the three joint moments, respectively.

**TABLE 1:** Two-stage approach with pre-training and fine-tuning for joint moment prediction.

Stage	Objective	Dataset	Label
Pre-Training^a^	Learn general spatiotemporal features from unlabeled lower-limb kinematics (Table 2)	18 kinematic features from the lower-limb	None
Fine-Tuning^b^	Predict knee, ankle, and hip moments from their labeled kinematics	6 joint angle–moment pairs	Each angle mapped to its corresponding moment

^a^ Utilizes masking and reconstruction to learn motion representations without force/moment labels.

^b^ Aligns specific lower-limb angles with labeled joint moments for supervised training.

## 2 Methods

### 2.1 Pre-training dataset

We used a joint angle dataset comprising 18 lower limb kinematic features, without labeled joint moments for our SSL pre-training. The dataset is based on a large gait analysis dataset (Senden et al., 2023), with typically developing children walking at different speeds.

The gait analysis dataset includes 55 typically developing children (24 boys and 31 girls) aged from 3 to 17 years (mean age 9.38 years, 95% CI: 8.51–10.25), with an average body mass of 35.67 kg (95% CI: 31.40–39.94) and leg length of 0.73 m (95% CI: 0.70–0.76). The dataset was specifically designed to capture substantial inter-subject variability and personalised walking patterns essential for detecting individual gait abnormalities. The significant anthropometric diversity, spanning a 14-year age range with considerable variation in body mass and leg length, naturally generates diverse joint moment patterns, as biomechanical loading varies substantially with body size, limb length, and developmental stage. For population stratification, participants were categorized into five age groups: 3–6 (n = 11), 7–8 (n = 10), 9–10 (n = 15), 11–12 (n = 11), and ≥13 years (n = 8), ensuring comprehensive representation across developmental stages, each characterized by distinct gait maturation patterns and joint loading characteristics. Participants performed walking tasks at three speeds defined as comfortable, 30% slower than comfortable, and 30% faster than comfortable in a virtual environment using the Computer Assisted Rehabilitation Environment (CAREN), including an instrumented treadmill and a 3D motion capture system with 12 cameras (Vicon Nexus v2.7) both operating at 100 Hz. For each speed condition, 250 gait cycles were recorded at a sampling frequency of 100 Hz. The dataset utilized 26 reflective markers placed at specific body landmarks according to the Human Body Lower Limb model with trunk markers (HBM2 (Motek BV, 2018), Figure 1) to capture gait kinematics during the walking task. A force plate (ForceLink, Culemborg, Netherlands) set at 15 Hz for the low-pass prefilter frequency and 20 N for the force threshold were used to measure the GRF during the walking task.

**FIGURE 1:**
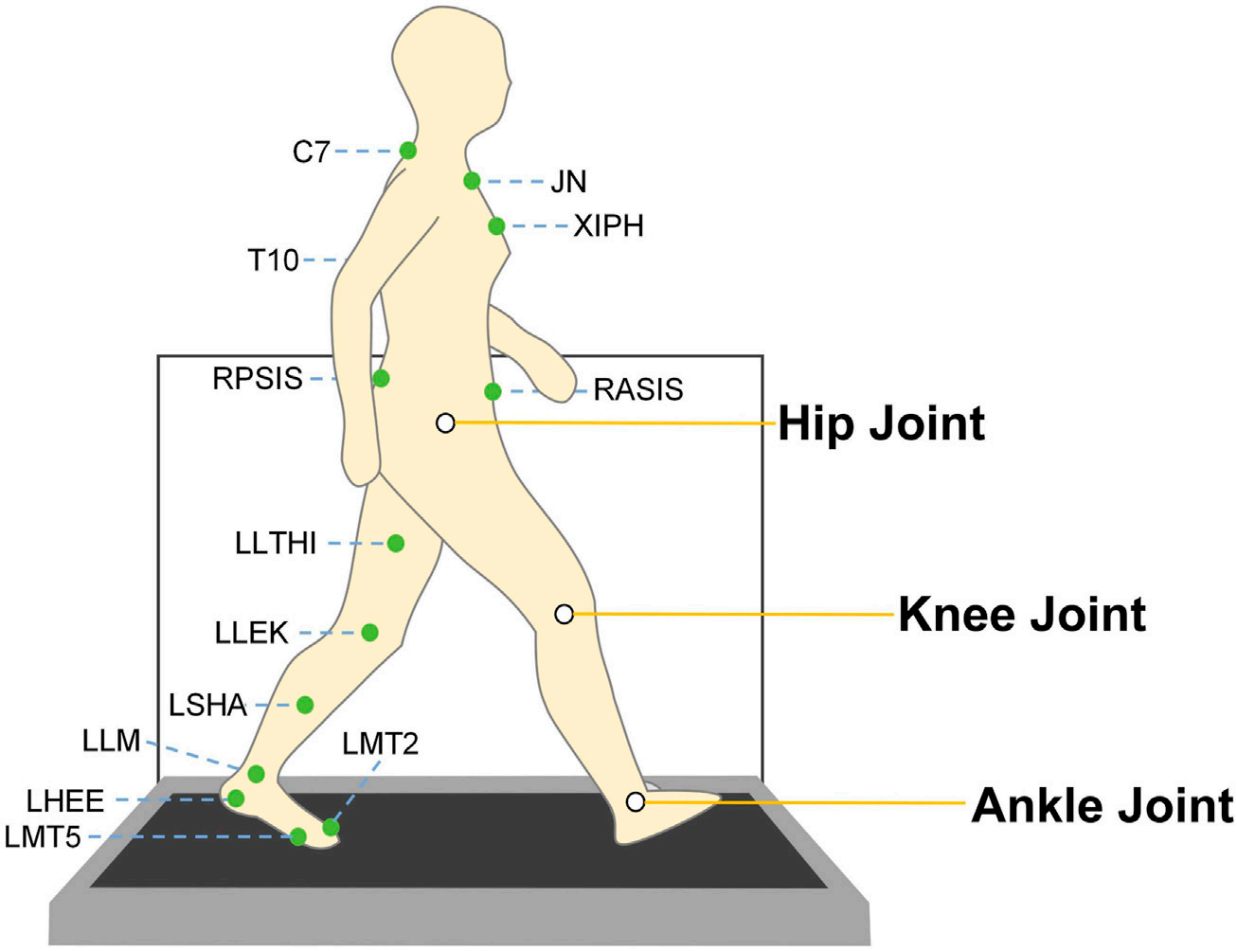
Illustration of marker points and major lower limb joint positions. The dataset (Senden et al., 2023) was acquired using the CAREN system, which integrates an instrumented treadmill equipped with force plates and a 12-camera three-dimensional motion capture system for comprehensive gait analysis. The green markers depicted in the figure represent the placement of reflective markers, which were positioned on prominent anatomical landmarks of the subject in accordance with the HBM2 (Human Body Model 2) protocol (Motek BV, 2018) The white circles in this figure highlight the primary joints of interest in this study—specifically, the hip, knee, and ankle joints. Kinematic data (joint and segmental angles) and kinetic data (joint moments) from these joints were extracted to facilitate the subsequent training and evaluation of deep learning models.

The raw data was processed using a low-pass filter with a cutoff frequency of 15 Hz. The GRF was normalized to body weight (Hof, 1996). All gait cycles were time-normalized from 0% to 100% of the gait cycle using linear interpolation, yielding 897 data samples. 18 kinematic features were extracted (Table 2). The processed data were represented as temporal waveforms on a uniform grid spanning 0%–100% of the gait cycle and extracted for subsequent training and evaluation.

**TABLE 2:** Overview of the 18 gait kinematics features for pre-training.

Joint name	Description
Lankleflex	Left ankle dorsiflexion/plantarflexion
Lanklepron	Left ankle pronation/supination
Lhipabad	Left hip abduction/adduction
Lhipflex	Left hip flexion/extension
Lhiprot	Left hip internal/external rotation
Lkneeflex	Left knee flexion/extension
Pelvicobl	Pelvic obliquity
Pelvicrot	Pelvic rotation
Pelvictil	Pelvic tilt
Rankleflex	Right ankle dorsiflexion/plantarflexion
Ranklepron	Right ankle pronation/supination
Rhipabad	Right hip abduction/adduction
Rhipflex	Right hip flexion/extension
Rhiprot	Right hip internal/external rotation
Rkneeflex	Right knee flexion/extension
Trunkflex	Trunk flexion/extension
Trunkrot	Trunk rotation
Trunktilt	Trunk tilt

### 2.2 Fine-tuning datasets

For our Fine-Tuning processing, we utilized six joint angle features, from the 18 kinematic features, and six corresponding joint moment datasets derived from the same gait analysis dataset (Senden et al., 2023). Joint moments were computed using inverse dynamics based on the inputs of marker trajectories and GRF, following the same pre-processing procedures applied to joint angles.

We extracted the LKneeFlex, RKneeFlex, LAnkleFlex, RAnkleFlex, LHipFlex, and RHipFlex angle datasets from the pre-training dataset and synchronized and merged each with its corresponding moment dataset based on time steps. The resulting restructured datasets (Table 1), which paired joint angles with their corresponding moments, were subsequently utilized in the fine-tuning phase.

### 2.3 Model architecture

**FIGURE 2:**
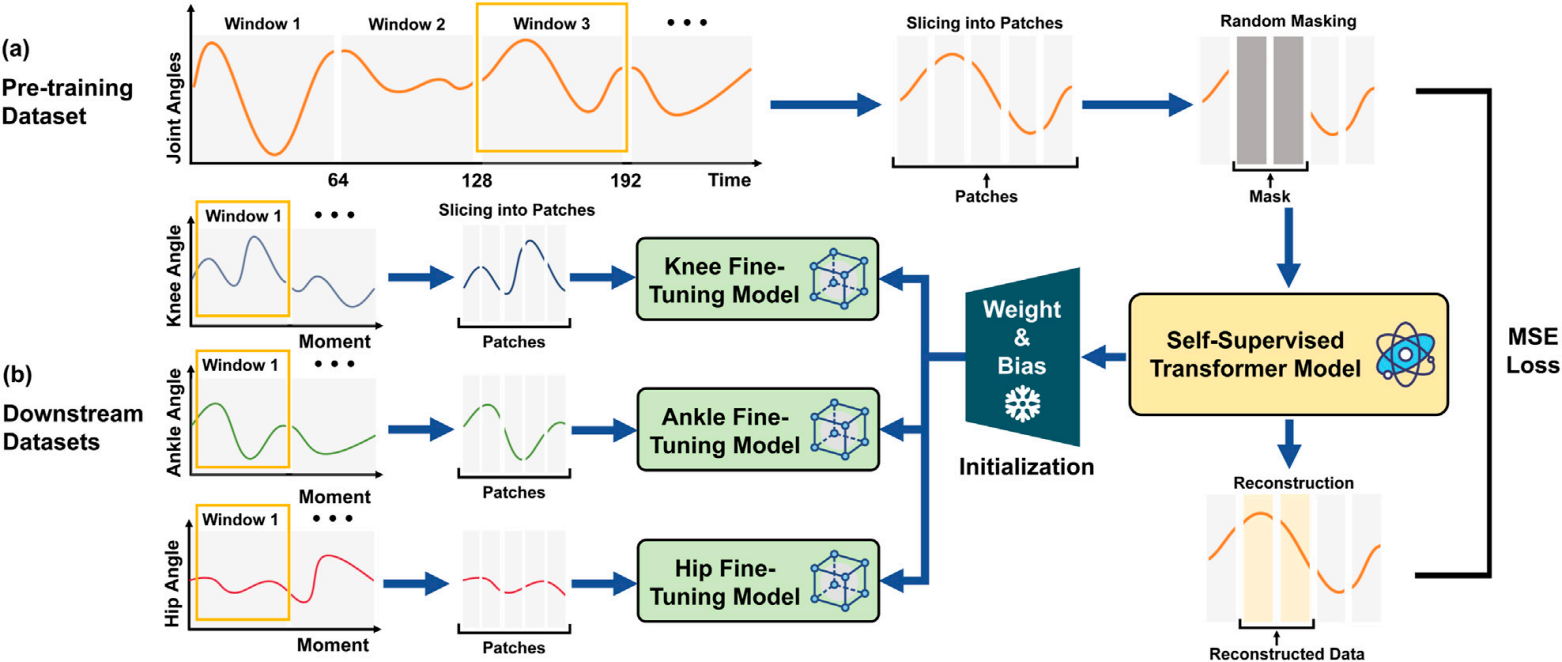
Framework of self-supervised learning for enhancing joint moment estimation from gait kinematics. **(a)** Pretraining Phase: Continuous time-series data are derived from angular measurements of 18 lower-limb kinematic features. These data are divided into overlapping time windows and subsequently segmented into fixed-length patches. A subset of the input fragments is randomly masked, and the resulting data are processed using a Transformer-based self-supervised model, which is trained by minimizing the reconstruction error through MSE loss. This process enables the model to effectively learn temporal dynamics and spatial correlations inherent in the data, thereby establishing a generalized representation of human motion. **(b)** Fine-tuning Phase: Utilizing the pre-trained model’s weights and biases as initialization, three separate fine-tuning models are developed to predict moments for the knee, ankle, and hip joints. The downstream data are preprocessed using the same windowing and patching approach as in the pre-training phase but without masking. The processed joint angular data are then fed into the respective fine-tuning models, with the objective of optimizing predictive performance for each specific task.

We developed a Transformer-based architecture to establish the relationship between joint angles and joint moments (Figure 2). The model comprises two stages: self-supervised pre-training and supervised fine-tuning.

We first used a Transformer auto-encoder (Figure 2a) to learn robust representations from large-scale unlabeled joint angle datasets. The Transformer’s input was a window of joint angle data with 64 samples with 18 kinematic features (Table 2). We then implemented a sliding window approach to segment the continuous joint angle data into fixed-length sequences of 64-time steps with an overlap of 1-time step between successive windows, resulting in a total of 33,189 windows. A 64-time-step window was selected to capture at least one complete gait cycle across most age groups and walking speeds, based on the average cycle duration observed in our dataset (typically 60–70 frames per step at 100 Hz), ensuring essential feature retention while avoiding redundancy that could increase the computational burden and reduce training efficiency. This fixed-length windowing strategy, combined with patch-wise encoding, allows the model to retain temporal locality within each gait cycle. The self-attention blocks further learn spatial correlations among joints at each time step, while the reconstruction task enforces the preservation of fine-grained biomechanical signals, enabling both local and global representations to be captured simultaneously. Then, the 64 samples were split into patches with a length of 1, maintaining input dimensions of [batch size, 64, 18], where 18 corresponds to the kinematic features listed in Table 2. After segmentation, 10% of the input data was randomly masked and replaced with a learnable mask embedding vector. Through comparative experiments with different masking ratios (5%, 10%, 15%, 20%) selected based on previous self-supervised learning studies (Kenton and Toutanova, 2019; Weng et al., 2024), we found that 10% offered the best balance for biomechanical data. We then randomly reordered the data before further analysis, minimizing potential biases introduced during data collection or pre-processing and ensuring a consistent distribution of the data. The model was trained to reconstruct the original masked data by minimising the MSE between the reconstructed and true inputs, with no explicit labels required for this self-supervised learning phase.

We linearly mapped each patch to a vector of length 24 and added sinusoidal positional encoding to preserve the temporal sequence information. The preprocessed data was then fed into the Transformer model, generating an output with dimensions identical to the input. The Transformer has eight self-attention blocks, with a per-block configuration of 12 attention heads, 2048 feedforward units, 10% dropout, $1\times 10^{-5}$ layer normalization, and rectified linear unit (ReLU) activation function. These hyperparameters were determined through a systematic grid search process. We experimented with various Transformer layers (4, 8, 12), attention heads (8, 12, 16), window sizes (32, 64, 128), and patch sizes (1, 2, 4) on the validation set, while maintaining consistent training configurations. The final configuration was selected based on the optimal balance between model performance (measured by reconstruction error) and computational efficiency. The outputs were linearly mapped back to the original data dimensions (patchlength×18jointfeatures) to reconstruct the unmasked input data, completing the auto-encoding process.

The downstream prediction task utilized a fine-tuning model built upon the pre-trained model (Figure 2b). This phase retained the core architecture and majority of the pre-trained parameters from the pre-trained model while fine-tuning the final layers to align with the new data distribution and prediction targets. We used the angles of the knee (left and right), ankle (left and right), and hip (left and right) as inputs with dimensions [batch size, 64, 2] for each joint-specific model, mapping the Transformer’s output to predict respective joint moments with corresponding output dimensions [batch size, 64, 2]. Similar to the joint angle pre-training process, we segmented the synthetic angle-joint mapped data into windows of 64 time steps with 1 time step overlap, obtaining 33,189 samples in total. Unlike pre-training, no masking was applied during the fine-tuning step, ensuring that the model could focus on task-specific prediction accuracy using supervised learning with MSE loss between predicted and labelled joint moments.

### 2.4 Training protocols

Models were implemented in Python 3.9 using PyTorch 1.9. An NVIDIA RTX 3070 Ti GPU was used to conduct the model training and testing process. The SSL pre-training phase required approximately 8.5 h of computational time, while the fine-tuning phase requiring only 2.8 h on average for each joint-specific model (knee, ankle, and hip) over 500 epochs. The total training time for our complete framework, including all six joint-specific fine-tuning models, was approximately 25 h.

We designed a two-stage approach (Table 1) to train the model: self-supervised SSL pre-training and supervised fine-tuning. This two-stage approach was designed to leverage large-scale unlabeled data effectively while ensuring accurate prediction of joint moments.

### 2.5 Self-supervised SSL pre-training

For the proposed SSL pre-training, we randomly masked a certain proportion of the time steps and trained the model to reconstruct the masked segments. We adopted a Transformer-based architecture due to its superior capability to capture long-range dependencies and parallel temporal processing, which are particularly advantageous in modeling complex gait dynamics. Reconstructing masked joint angles helps the model capture both local continuity and global coordination patterns that are essential for downstream tasks such as joint moment prediction (Zhang et al., 2024).

We utilized the Adam optimizer (Kingma, 2014) to adaptively adjust learning rates based on the first and second moments of gradients, using an initial learning rate of $1\times 10^{-4}$ and batch size of 64. The model was trained for 2000 epochs with the learning rate adjusted using a cosine annealing schedule (Loshchilov and Hutter, 2016) to facilitate smooth and effective convergence. We employed the entire dataset for pre-training without setting aside a separate test set, as the model evaluation was exclusively carried out on downstream datasets that were different from those used during pre-training. To mitigate overfitting, we applied weight decay (Krogh and Hertz, 1992) at a rate of $1\times 10^{-5}$ and incorporated dropout (Srivastava et al., 2014) with a rate of 0.1. These regularization techniques ensured improved generalisation by penalizing large weights and reducing reliance on specific neurons during training.

### 2.6 Supervised fine-tuning

For downstream evaluation, we employed a fine-tuning strategy to adapt the pre-trained model to the specific task of joint moment estimation. Joint flexion and extension features were used as inputs during fine-tuning, as these movements represent the primary planar motions in the sagittal plane during gait and contribute most significantly to the lower-limb joint loading patterns (Gray et al., 2019). Additionally, flexion-extension movements typically show higher signal-to-noise ratios and greater reproducibility across participants compared to other features.

We varied the proportion of training samples used for fine-tuning, ranging from 5% to 100% of the fine-tuning dataset. Unlike the pre-training process, we split the dataset into the training, validation, and test sets in proportions of 70%, 20%, and 10%, respectively. We first froze 75% of the pre-trained model’s parameters by freezing the first six layers and unfreezing the final two layers, which was empirically validated in our preliminary experiments to provide the best trade-off between generalization and task-specific adaptation, ensuring that the foundational knowledge extracted from the pre-training process remained intact. This parameter freezing strategy is particularly important for Transformer models, which are known to suffer from catastrophic forgetting during transfer learning. By limiting updates to only the higher layers, the model can better retain generalizable motion representations while adapting to the downstream angle-to-moment mapping task.

To ensure robust performance and prevent overfitting when working with limited labeled data, we implemented comprehensive monitoring and mitigation strategies throughout the fine-tuning process. We employed early stopping based on validation loss with a patience of 20 epochs to automatically terminate training when no improvement was observed, preventing excessive adaptation to small training sets. Throughout the fine-tuning process, we continuously monitored the gap between training and validation losses to ensure consistent generalisation performance. The SSL pre-training phase itself provides inherent regularisation benefits by establishing robust spatiotemporal representations from large-scale unlabeled data, creating strong inductive biases that naturally resist overfitting during fine-tuning. Combined with our unified regularization parameters (weight decay of $1\times 10^{-5}$ and dropout rate of 0.1 maintained consistently across all data scenarios) and parameter freezing strategy, these mechanisms enabled reliable performance even when fine-tuning with as little as 5% of labelled data.

Then, we fine-tuned the weights and biases based on the mapping between the angles and moments of the knee, ankle, and hip (Yosinski et al., 2014). This approach helps preserve the general, domain-invariant representations learned by the pre-trained model, mitigates overfitting, and reduces the training complexity and computational costs, improving both efficiency and stability. We utilized the Adam optimizer with a lower initial learning rate of $1\times 10^{-6}$ and batch size of 64. To preserve the representations acquired during pre-training, we set the initial fine-tuning learning rate to be 100 times smaller than that used in the pre-training phase. The model was trained for 500 epochs with the learning rate adjusted using a Plateau-Based Learning Rate schedule to dynamically adjust the learning rate based on the validation set loss.

### 2.7 Baseline model

The baseline model functions as a comparative benchmark to assess the effectiveness of the proposed pre-trained SSL and fine-tuning approach.

We constructed the baseline model utilizing an identical model architectural framework as the proposed pre-trained SSL model but trained it directly on a fully labeled dataset (labeled joint moment for each angle in the pre-training dataset) without any pre-training using SSL. To ensure a fair comparison, we employed the same optimizer and learning rate strategy as the SSL pre-training approach.

The baseline model has demonstrated strong performance in joint moment estimation tasks and thus serves as a suitable benchmark for evaluating the effectiveness of the proposed model.

### 2.8 Performance evaluation

We utilized MSE (Equation 1) and MAE (Equation 2) (Karatsidis et al., 2018) to evaluate the effectiveness of the proposed SSL and fine-tuning approach, which are defined as follows:

$$\text{MSE}=\frac{1}{N}\overset{N}{\underset{i=1}{\sum }}{\left(y_{i}-{\hat{y}}_{i}\right)}^{2}$$ (1)

$$\text{MAE}=\frac{1}{N}\overset{N}{\underset{i=1}{\sum }}\left|y_{i}-{\hat{y}}_{i}\right|$$ (2)

where N denotes the total number of samples, $y_{i}$ denotes the actual value for the i -th sample, and ${\hat{y}}_{i}$ denotes the predicted value for the i -th sample.

To ensure the robustness and reliability of our results, each experimental condition was evaluated across 10 independent training runs using different random seeds to account for the inherent variability in deep learning model training. The performance metrics reported throughout this study represent the mean performance across these multiple runs.

We employed a comprehensive evaluation strategy to assess the performance of the proposed model, focusing on two main aspects: prediction accuracy and data efficiency. By incorporating both MSE and MAE metrics, we can effectively evaluate the model’s accuracy and robustness.

### 2.9 Prediction accuracy

Prediction accuracy is a crucial metric for evaluating model performance, as it enhances the reliability and applicability of the model while reducing the risks of misdiagnosis and incorrect assessments.

We conducted a comprehensive comparative evaluation of the proposed model and the baseline Transformer model using MSE and MAE, indicating the accuracy and stability of the model. Furthermore, we extended our analysis to include a detailed examination of the errors between the predicted values produced by the proposed model, the baseline Transformer model, and the true values on the test set at each time step, assessing the model performance dynamically. This highlighted the performance differences under various conditions and provided deeper insights into the models’ prediction accuracy.

### 2.10 Data efficiency

Data efficiency is a critical aspect of modern machine learning, particularly in scenarios characterized by large-scale data requirements or data scarcity (Chen et al., 2020a; Grill et al., 2020). It focuses on optimizing model performance while minimizing resource consumption. Data efficiency enables models to achieve competitive performance without requiring proportional increases in data volume. It is particularly advantageous for high-dimensional and complex data, where labeleding costs are prohibitive or the availability of labeled samples is inherently limited.

We simulated data scarcity by varying the amount of training data used during the fine-tuning stage while maintaining a fixed test set. We trained the model using varying proportions of the fine-tuning dataset to simulate different levels of data availability. The performance of the fine-tuned models was assessed using MSE, and the results were compared to the baseline model. The findings can demonstrate the potential of SSL pre-training to reduce dependency on labeled data while maintaining or improving predictive accuracy.

Furthermore, we conducted an extensive investigation into the relationship between input-output configurations and prediction performance through additional experimentation. We utilized the angle features of the three joints as the sole input to predict each of their respective joint moments. This setup was designed to evaluate the model’s capacity to predict multiple joint moments from single-joint input and to explore inter-joint motion relationships.

## 3 Results

### 3.1 Prediction accuracy

The proposed pre-trained and fine-tuned model achieved higher accuracy in estimating joint moments based on corresponding joint angles compared to baseline models with identical architecture.

**FIGURE 3:**
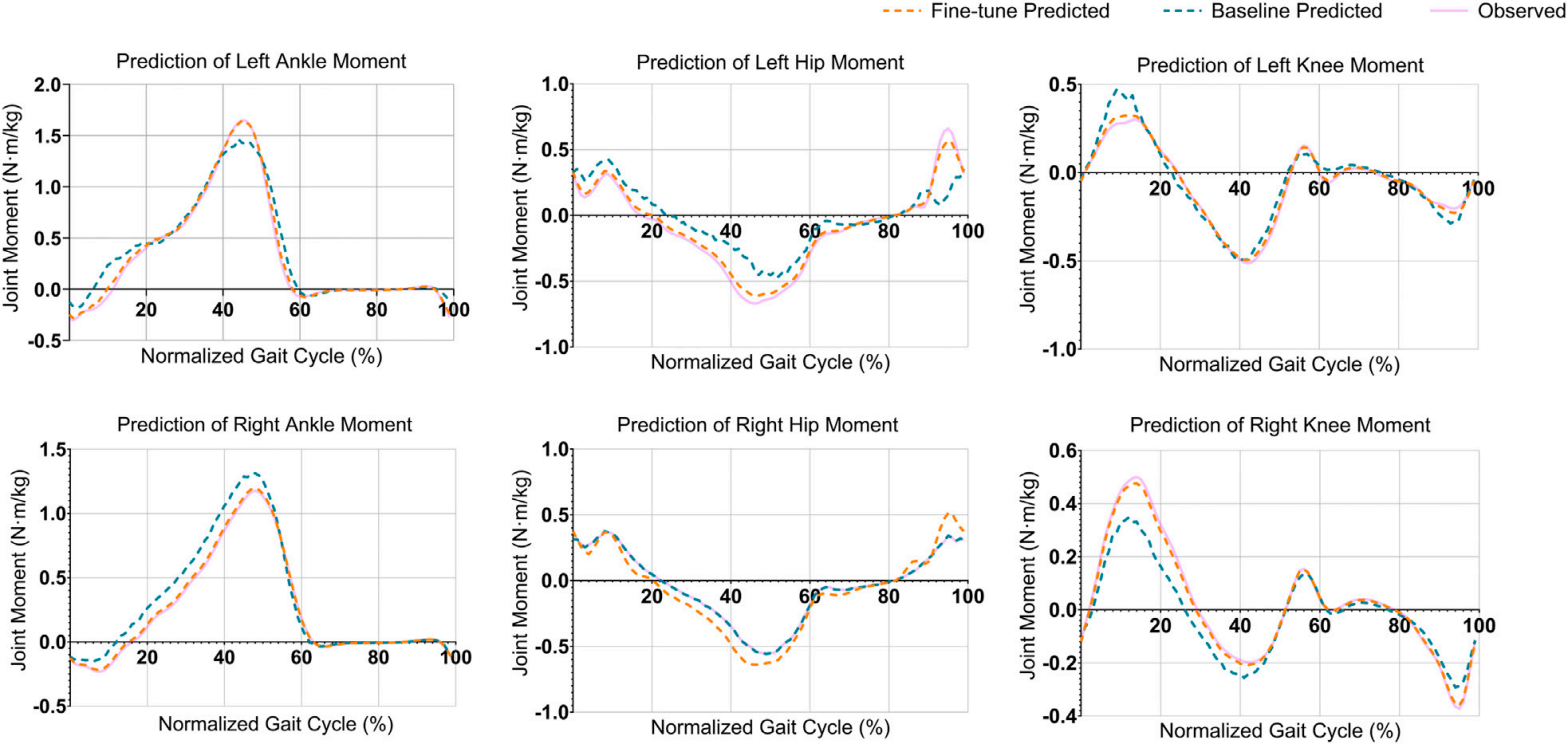
Comparison of joint moment predictions across six joints during a normalized gait cycle. Each plot represents the predicted and observed joint moments (in N⋅m/kg ) for a specific joint, including the left and right knee, ankle, and hip. The dashed orange lines indicate predictions from the fine-tuned model, the dashed green lines represent predictions from the baseline model, and the solid purple lines show the observed joint moments. The fine-tuned model consistently aligns closer to the observed joint moments, demonstrating improved prediction accuracy, especially in capturing dynamic trends throughout the gait cycle.

The alignment among peak joint moments (indicating maximum load or muscle activation) and valley joint moments (indicating minimum load or muscle activation) improved significantly with the proposed model, achieving improvements ranging from 50% (15% gait cycle of the left knee) to 100% (50% gait cycle of the left ankle) across multiple joints (Figure 3). With the fine-tuned model, the average deviation at these peaks was roughly 0.02 N⋅m/kg , compared to about 0.04 N⋅m/kg for the baseline of 50% reduction in error. Similarly, during valley phases (e.g., mid-stance, occurring within the 0%–60% gait cycle range), the proposed model lowered errors from around 0.03 to 0.015 N⋅m/kg . Among the three joints, the prediction of the LHipFlex and RHipFlex moment demonstrated the best optimization, especially when the moments exhibit a gradual change.

To address the clinical utility requirements, we conducted a focused analysis of prediction errors at peak joint moments, which represent the most clinically relevant phases of the gait cycle for biomechanical assessment. The fine-tuned model demonstrated superior accuracy at these critical moments across all joints. For the knee joints, the mean absolute error at peak flexion moments was 0.015 ± 0.006 N ⋅ m/kg for the left knee and 0.011 ± 0.006 N ⋅ m/kg for the right knee, representing relative errors of approximately 7.5% and 2.9% of the true peak moments, respectively. The ankle joints showed exceptional performance with mean absolute errors of 0.014 ± 0.008 N ⋅ m/kg (left) and 0.009 ± 0.006 N ⋅ m/kg (right) at peak plantarflexion moments, corresponding to remarkably low relative errors of 5.2% and 1.1% respectively. Hip joints demonstrated mean absolute errors of 0.081 ± 0.049 N ⋅ m/kg (left) and 0.048 ± 0.024 N ⋅ m/kg (right) at peak extension moments, with relative errors of 12.9% and 8.7%. These peak-specific error metrics demonstrate that the model maintains high accuracy during the most critical phases of gait.

**FIGURE 4:**
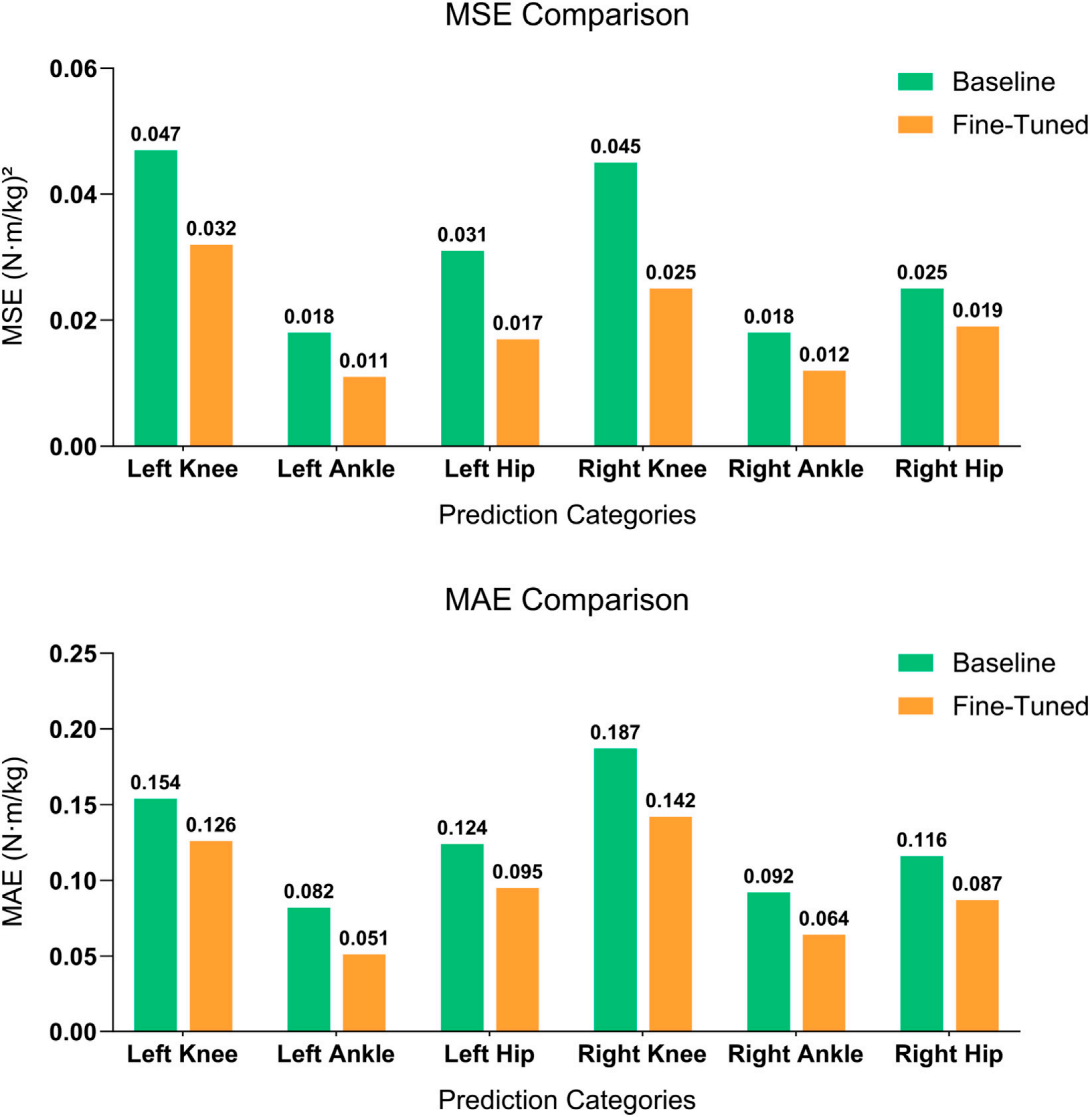
Comparison of MSE (top) and MAE (bottom) between baseline model and fine-tuned model. Models for joint moment prediction based on the corresponding joint angle across six joints: left knee, left ankle, left hip, right knee, right ankle, and right hip. The fine-tuned model demonstrates significant improvements in both metrics across all categories, highlighting its superior performance in predicting joint moments with reduced error.

The predictions of different joint moments demonstrated overall improvements when analyzing the MSE and MAE (Figure 4). The LAnkleFlex and RAnkleFlex moments predictions showed the most precise results with MAE values of 0.051 N⋅m/kg and 0.064 N⋅m/kg , respectively, improving by 37.8% and 30.4% over the baseline. LAnkleFlex moments estimated by the fine-tuned model demonstrated the lowest MSE (0.011 ${\left(N\cdot m/kg\right)}^{2}$ ), reflecting an improvement of 38.9% compared to the baseline. Similarly, the RAnkleFlex moments also performed exceptionally well with the MSE reducing to 0.012 ${\left(N\cdot m/kg\right)}^{2}$ , a 33.3% improvement over the baseline. Additionally, the MSE was reduced by approximately half for the RKneeFlex moments prediction (44.4%) and LHipFlex moments prediction (45.2%). The MAE of the RKneeFlex moment prediction also reduced by 24% from 0.187 to 0.142 N⋅m/kg .

### 3.2 Data efficiency

**FIGURE 5:**
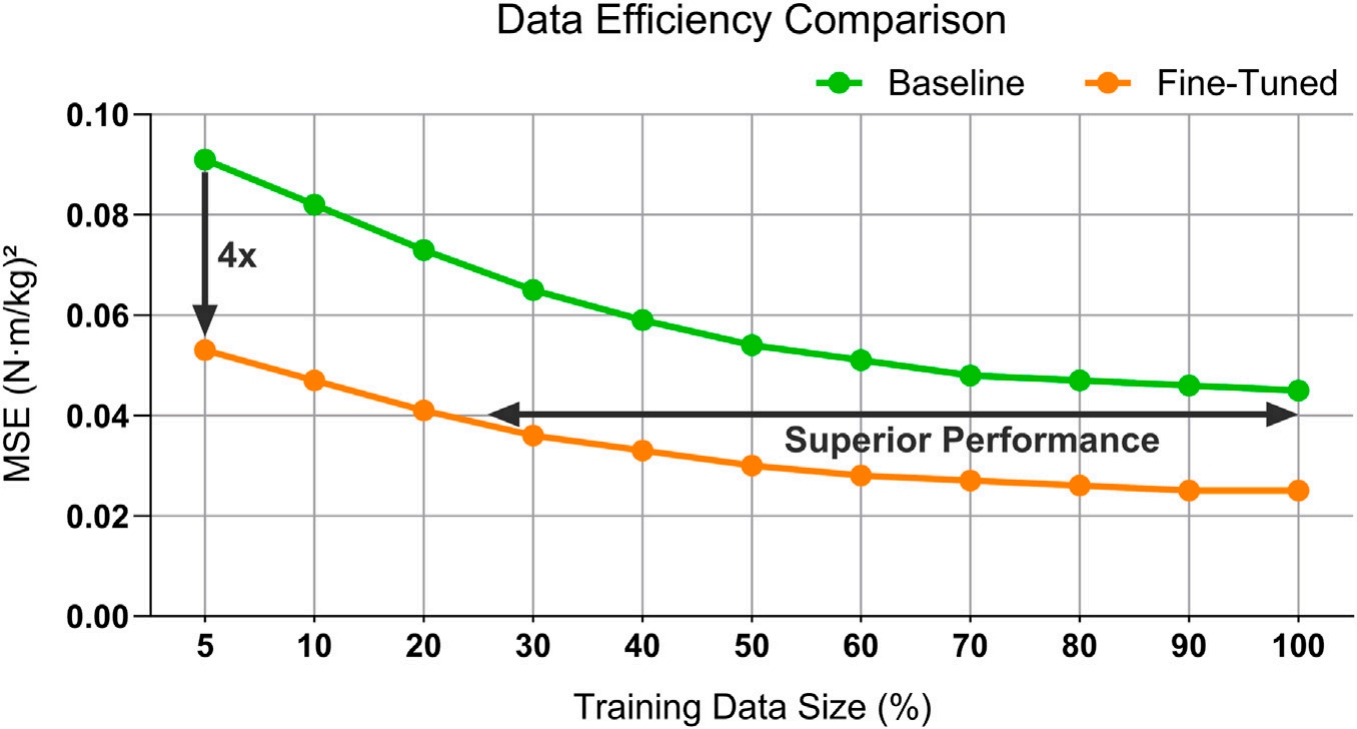
Comparison of data efficiency between the baseline model and the fine-tuned model for the right knee moment prediction. The fine-tuned model demonstrates consistently superior performance across varying labeled training data sizes, achieving lower MSE and highlighting the benefits of the pre-training and fine-tuning framework.

The fine-tuned model consistently outperforms the baseline, achieving significant error reductions across all training data sizes, and highlighting its superior data efficiency and predictive accuracy (Figure 5).

When fine-tuned with only 20% of the training data, the proposed SSL model achieved a right knee MSE of approximately 0.041 ${\left(N\cdot m/kg\right)}^{2}$ , matching—or even slightly surpassing—that of the baseline model trained on the full dataset. Increasing the labeled data to 50% allowed the SSL model to further reduce the MSE of the right knee to roughly 0.030 ${\left(N\cdot m/kg\right)}^{2}$ , a 44.44% improvement over the baseline of 0.054 ${\left(N\cdot m/kg\right)}^{2}$ . Even under more extreme constraints, using only 5% labeled data, the SSL approach can maintain an MSE of around 0.053 ${\left(N\cdot m/kg\right)}^{2}$ , a roughly four-fold decrease compared to the baseline of 0.091 ${\left(N\cdot m/kg\right)}^{2}$ , highlighting the robustness and data efficiency gained through self-supervised pre-training.

### 3.3 Other results

**FIGURE 6:**
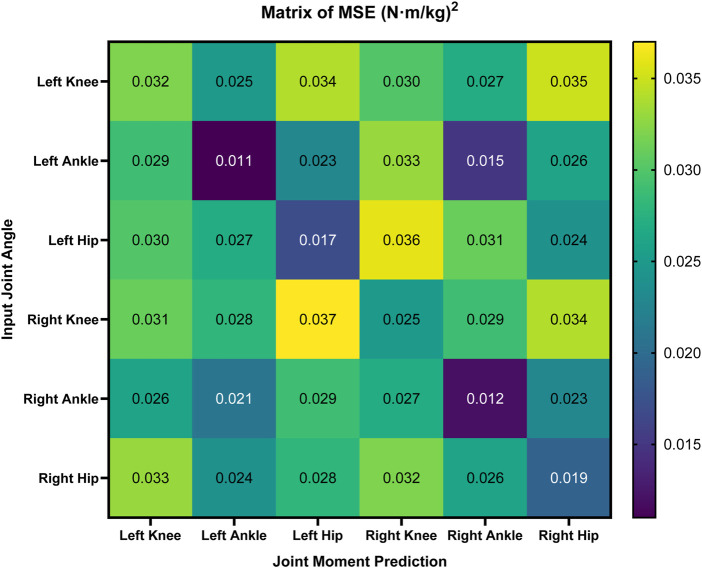
Matrix of MSE for joint moment prediction across different input joint angles. The MSE values $\left({\left(N\cdot m/kg\right)}^{2}\right)$ demonstrate the prediction performance for each joint combination, with darker shades indicating lower errors and brighter shades representing higher errors. This visualization aids in identifying the relative accuracy of predictions across various input-output joint pairings.

We designed a matrix (Figure 6) to demonstrate the MSE for predicting the six joints’ moments from each joint’s angles, revealing both the strong local correlations in same-joint mappings and the inter-joint synergy captured in cross-joint predictions.

In particular, diagonal cells, representing the same joint mappings such as the angle of the left ankle to the moment of the left ankle, generally exhibit the lowest errors (e.g., around 0.010 to 0.020 ${\left(N\cdot m/kg\right)}^{2}$ , confirming that local angular data are the strongest predictors of local joint moments. For the same joint mappings, our proposed model shows a consistent reduction in MSE compared to the baseline model (e.g., 0.011 vs. 0.018 ${\left(N\cdot m/kg\right)}^{2}$ for left ankle, a 38.9% reduction). Off-diagonal cells, such as estimating the right knee moment from the left knee angle, show moderately higher MSE (e.g., approximately 0.028–0.037 ${\left(N\cdot m/kg\right)}^{2}$ . The model’s ability to maintain reasonable accuracy in cross-joint scenarios highlights the robust internal representations it learned during self-supervised pre-training, indicating enhanced generalization for more complex prediction tasks.

## 4 Discussion

This work demonstrated that using SSL to pre-train the Transformer model for estimating joint moments from joint angles can improve prediction accuracy and data efficiency. Previous DL models for biomechanical estimation required large-scale datasets with completely manually labeled data and extensive laboratory time for data collection. In contrast, we used a limited amount of labeled data to establish the relationship between different joint angles and moments. From a practical implementation perspective, while our SSL approach requires a one-time computational investment of approximately 25 h, traditional biomechanical data collection for generating equivalent labelled datasets would require approximately 100–200 h of laboratory time, including participant recruitment, experimental setup, data collection, and post-processing. Moreover, our pre-trained model can be efficiently fine-tuned for new populations or conditions in less than 3 h, compared to weeks or months required for collecting new labelled datasets in clinical settings. Three key outcomes of this work are:

• Improved prediction accuracy and data efficiency: Compared to supervised learning, the SSL pre-trained model can improve data efficiency by more than 80%. Furthermore, it can improve prediction accuracy by more than 40% when training with the same amount of labeled data.

• Discovering the spatiotemporal representations between different joints and movements: The use of a 10% masking ratio during pre-training led to enhanced performance, revealing the inherent structure and regularity of lower-limb joint movements. Additionally, the prediction accuracy of joint moments was evaluated using various joint angles, highlighting the interrelation among lower-limb joints.

• Proven scalable framework: The two-stage approach provides the flexibility of expanding the model to additional joints, tasks, or populations, enabling broader applications in clinical and biomechanical assessments.

In recent studies, joint kinematics, such as joint angles, combined with electromyography (EMG) signals, have been widely utilized to predict joint moments (Zhang et al., 2022a; Wang et al., 2023; Serbest et al., 2023). In this work, we focus exclusively on joint angles as input features for moment prediction, simplifying data collection and assessing the feasibility of achieving accurate predictions without additional features. Furthermore, we broaden the scope of joint moment prediction by including a more diverse set of joint angles and movements, enabling the model to capture complex relationships between joint kinematics and their corresponding joint moments. This approach aims to uncover nuanced interactions, providing a more comprehensive representation of human biomechanics.

Additionally, two-stage approaches leveraging self-supervised pre-training followed by supervised fine-tuning have been widely explored in fields such as vital sign monitoring (Yfantidou et al., 2024), image analysis (Feng et al., 2021), and adversarial learning (Chen et al., 2020b; Zakarias et al., 2024). However, their application in biomechanical modeling remains relatively unexplored, where conventional approaches still predominantly rely on fully supervised learning with large amounts of labeled data—resources that are often difficult and expensive to obtain. In this study, we adapt and validate a two-stage self-supervised learning paradigm within the angle-to-moment time-series domain for the first time, demonstrating its potential to improve predictive accuracy while substantially reducing reliance on labeled datasets. Due to the absence of publicly available benchmark models trained on the same dataset, it was not feasible to perform a fair comparison across different SSL strategies. To ensure a controlled evaluation, we compared our method against a baseline model with an identical Transformer architecture trained from scratch. The results confirm that, by leveraging large-scale unlabeled joint kinematic data, our approach effectively captures spatiotemporal representations and improves generalization performance, presenting a practical alternative to conventional supervised pipelines in biomechanical modeling.

SSL trains a model to capture the relationships between motions of different joint angles specific to the pre-training data. Consequently, it yielded the most significant improvement in joint moment estimation for the knee joint. Specifically, compared to baseline model trained from scratch, our fine-tuned model achieved a 44% reduction in right knee MSE (from 0.045 to 0.025) and a 32% reduction in left knee MSE (from 0.047 to 0.032). Notably, using only 20% of the labeled data, our SSL-based model achieved comparable performance to a fully supervised model trained with 100% of the labeled data, demonstrating the data efficiency of SSL. These substantial gains in knee prediction may indicate that the knee experiences greater loading variations during the gait cycle, providing richer spatiotemporal cues for the model to leverage (Telfer et al., 2017). also supported this observation, highlighting that knee joint moments exhibit notable variability influenced by specific kinematic and spatiotemporal parameters, including gait speed, stance duration, and knee flexion angles, thus making these parameters particularly sensitive for knee moment prediction.

Although the ankle and hip joints also benefited from pre-training, their improvements were comparatively smaller. For example, the left ankle MSE dropped from approximately 0.018 to 0.011 (a 39% reduction), and the right ankle from 0.018 to 0.012 (33%). For the hip, the left joint MSE was nearly halved, decreasing from 0.031 to 0.017, while the right hip saw a more modest reduction from about 0.025 to 0.019 (24%).

These results confirm that even for joints where baseline error metrics were relatively low, the pre-trained representations enhanced predictive performance. Moreover, the reduction in MSE highlights a decrease in large error instances, while the MAE reflects the model’s overall performance by capturing the average magnitude of errors. The notable decline in MSE, alongside a modest MAE reduction, indicates that the proposed model achieves not only lower overall error but also greater consistency and stability across different joint predictions.

The significantly improved prediction of peak and valley joint moments during a normalised gait cycle demonstrates the proposed model’s superior ability to capture dynamic features and accurately predict time series data (Zhang et al., 2024; Kenton and Toutanova, 2019). This highlights its strong spatiotemporal modelling capability. From a clinical perspective, such accuracy in detecting joint-loading extremes is crucial for identifying pathological gait patterns associated with conditions like knee osteoarthritis or neuromuscular disorders. Shifts in peak and valley moments often serve as indicators of progressive joint deterioration or compensatory strategies (Miyazaki et al., 2002; Delp et al., 2007), making this level of accuracy invaluable for early diagnosis and intervention.

One of the most compelling advantages of the proposed SSL-based framework is its ability to achieve high predictive accuracy while significantly reducing reliance on labeled data. Our results demonstrate that the SSL model can match—or even outperform—the baseline model trained from scratch with 100% labeled data, using only 20% of the labeled dataset for fine-tuning. Even under more extreme constraints—such as fine-tuning with only 10% of the labeled data—the SSL model’s MSE remains below 0.050, reflecting up to a fourfold improvement over the baseline.

This level of data efficiency underscores the value of self-supervised pre-training in offering a strong initialization that facilitates effective adaptation with limited labeled data. Beyond reducing the cost and time required for experimental data collection, this also alleviates participant burden, particularly for populations that are difficult to access or at higher risk (e.g., paediatric or clinical cohorts). Notably, the baseline model’s performance deteriorates sharply when access to labeled examples is restricted, with the MSE nearly doubling from 0.045 to 0.100 ${\left(N\cdot m/kg\right)}^{2}$ ). In contrast, the SSL model maintains low error by leveraging the robust spatiotemporal representations learned during pre-training, demonstrating the potential of self-supervised learning to drastically reduce the need for extensive labeled datasets while preserving—or even enhancing—predictive accuracy compared to models trained from scratch.

The trend of the cross-joint predictions (Figure 6) demonstrated that each joint’s moment is predominantly driven by its angular variations, although inter-joint coupling (e.g., knee–ankle synergy) also influences dynamic consistency. Interestingly, the matrix further reveals that certain cross-joint predictions can nearly match the accuracy of same-joint mappings. For instance, predicting the right ankle moment from the left ankle angle yields an MSE of around 0.015 ${\left(N\cdot m/kg\right)}^{2}$ , only marginally higher than the 0.012 observed on the diagonal (i.e., right ankle angle to right ankle moment). This outcome highlights the fine-tuned Transformer model’s ability to capture multi-joint synergies in gait, leveraging spatiotemporal representations learned from a large corpus of unlabeled angle data. The model appears to leverage subtle inter-limb correlations—for instance, a compensatory modification in ankle motion if knee mobility is constrained. Such insights hold clinical relevance, as the improved understanding of how movements at one joint influence loading patterns at another joint could guide rehabilitation professionals in designing more targeted interventions, especially for conditions involving compensatory gait strategies (Nadeau et al., 1997). has shown that individuals with Patellofemoral Pain Syndrome (PFPS) exhibit lower knee extensor moments (0.104 N⋅m/kg , 16% lower) and higher hip extensor moments (0.064 N⋅m/kg , 56% higher) during the early stance phase compared to healthy controls. Given that our SSL model can accurately capture joint moment variations with a MSE as low as 0.015 ${\left(N\cdot m/kg\right)}^{2}$ in cross-joint predictions, it demonstrates the potential to distinguish such subtle biomechanical differences in clinical assessments. This suggests that our approach may aid in the early detection of PFPS and other subtle gait abnormalities, enabling timely interventions and treatment adjustments.

In addition, the bilateral symmetry observed in cross-joint predictions has direct implications for rehabilitation protocol design, suggesting that unilateral training interventions may have predictable effects on the contralateral limb, supporting the clinical practice of using bilateral exercises for unilateral deficits. Additionally, the cross-joint prediction capabilities demonstrate potential for advancing prosthetic control systems, where predicting joint moments from kinematic data of intact joints could inform the development of more intuitive prosthetic devices that anticipate loading requirements based on residual limb movements.

## 5 Limitations and future work

Several limitations of this study provide important directions for future research.

### 5.1 Data expansion

While our study demonstrates the effectiveness of SSL in a pediatric population (3–17 years), its generalisation to broader demographics remains an open challenge. Adults and elderly individuals exhibit distinct anthropometric profiles, slower gait speeds, and increased joint stiffness, all of which may affect the learned angle-to-moment mappings. Moreover, pathological populations such as those with cerebral palsy, stroke, or Parkinson’s disease often develop individualized compensatory strategies that fall outside the current training distribution.

To address these limitations, we plan to leverage the demonstrated data efficiency of our SSL framework through transfer learning and domain adaptation techniques. Specifically, pre-trained models will serve as initialisations for fine-tuning on smaller, population-specific datasets. This approach is particularly beneficial in clinical contexts, where acquiring large labeled datasets from elderly or pathological populations poses ethical and practical challenges.

In future work, we will systematically validate the proposed SSL framework across different age groups and clinical cohorts. We also aim to diversify the training dataset to include additional movement types—such as running, jumping, and cutting—to test the model’s adaptability across cyclic and non-cyclic motor patterns (Giarmatzis et al., 2015). These expansions will further assess the robustness and generalizability of our approach beyond walking.

### 5.2 Technical improvements

While our SSL-based model achieves high prediction accuracy and strong data efficiency, its current computational footprint has not been optimized for real-time deployment. This limitation may hinder practical adoption in wearable and mobile health applications, where immediate feedback is critical for continuous biomechanical monitoring.

To address this, future work will focus on real-time implementation optimization through advanced model compression techniques, including knowledge distillation, post-training quantization, and structured pruning. These approaches aim to reduce inference latency and memory demands without compromising predictive performance. The ultimate goal is to enable seamless integration with wearable IMU systems and edge devices, supporting real-world gait analysis and joint moment estimation outside laboratory settings.

### 5.3 Data analysis

While our study demonstrates the effectiveness of SSL for joint moment estimation and highlights its potential for improving data efficiency, it does not include comprehensive comparisons with conventional biomechanical modeling methods or alternative deep learning architectures. This limits our ability to conclusively establish the relative advantages of our approach within the broader methodological landscape.

To address this limitation, future research will include systematic cross-paradigm evaluations against established models such as inverse dynamics-based biomechanical estimators, recurrent architectures like LSTMs for temporal sequence modelling, CNNs for spatial feature extraction, and hybrid CNN-LSTM frameworks that integrate both.

In addition, we plan to conduct formal statistical power analyses and larger-scale validation studies with predefined sample size calculations. These efforts will enhance the statistical robustness of our findings and support their translational potential in clinical and real-world applications.

## 6 Conclusion

This study demonstrates that self-supervised learning (SSL) can substantially enhance both the accuracy and data efficiency of lower-limb joint moment estimation from gait kinematics. By leveraging large-scale, unlabeled joint angle datasets, our framework achieved up to 36% improvements in MSE while requiring only 20% of the labelled data to match the performance of fully supervised models. These findings highlight the potential of SSL to reduce reliance on costly labelled data and accelerate the deployment of biomechanical models in clinical and field-based settings. Our work provides a scalable and data-efficient foundation for future joint load estimation systems, offering a promising step toward accessible, real-time gait assessment beyond laboratory environments.

## Data Availability

The datasets presented in this study can be found in online repositories. The names of the repository/repositories and accession number(s) can be found below: https://osf.io/3xqew/.
